# The FACAM study: protocol for a randomized controlled study of an early interdisciplinary intervention to support women in vulnerable positions through pregnancy and the first 5 years of motherhood

**DOI:** 10.1186/s13063-022-06022-4

**Published:** 2022-01-24

**Authors:** Maiken Pontoppidan, Lene Nygaard, Mette Thorsager, Mette Friis-Hansen, Deborah Davis, Ellen Aagaard Nohr

**Affiliations:** 1 VIVE – The Danish Centre for Social Science Research, Herluf Trolles Gade 11, 1052 Copenhagen, Denmark; 2 University of Canberra and ACT Health, Bruce, Australia; 3 Research Unit for Gynecology and Obstetrics, Department of Clinical Research, University of Southern Denmark, Odense, Denmark; 4 Department of Gynaecology and Obstetrics, Odense University Hospital, Odense, Denmark

**Keywords:** Pregnant, Mother, Mental health, Support, Multidisciplinary, Early intervention, Vulnerable families, Disadvantaged populations, Poverty, Parent, Substance use, High risk, Randomized controlled trial, Qualitative

## Abstract

**Background:**

Inequality in health can have profound short- and long-term effects on a child’s life. Infants develop in a responsive environment, and the relationship between mother and infant begins to develop during pregnancy. The mother’s ability to bond with the fetus and newborn child may be challenged by mental health issues which can cause impaired functioning and poorer health outcomes. Families with complex problems need interdisciplinary interventions starting in early pregnancy to be prepared for motherhood and to ensure healthy child development. This study aims to examine the effects of an early and coordinated intervention (the Family Clinic and Municipality (FACAM) intervention) offered to vulnerable pregnant women during pregnancy and the child’s first year of life on the mother-child relationship, maternal social functioning, mental health, reflective functioning, well-being, parental stress, and the development and well-being of the child.

**Methods:**

The study is a prospective randomized controlled trial where we will randomize 320 pregnant women enrolled to receive antenatal care at the family clinic at Odense University Hospital, to either FACAM intervention or usual care. The FACAM intervention consists of extra support by a health nurse or family therapist during pregnancy and until the child starts school. The intervention is most intensive in the first 12 months and also includes attachment-based support provided either individually or in groups. The participants are assessed at baseline, and when the infant is 3 and 12 months old. The primary outcome is maternal sensitivity measured by the Coding Interactive Behavior (CIB) instrument. Secondary outcomes include prenatal parental reflective functioning, mental well-being, depressive symptoms, breastfeeding duration, maternal satisfaction, child development, parent competence, parental stress, and activities with the child.

**Discussion:**

The trial is expected to contribute knowledge about the effect of early coordinated support in antenatal and postnatal care for vulnerable pregnant women and their families.

**Trial registration:**

ClinicalTrials.gov NCT03659721. Registered on September 6, 2018

## Background

Pregnancy and the transition to becoming a parent are characterized by rapid psychological, physiological, and social changes, which can be challenging for both the mother, father, and infant. Parents with reduced resources (psycho-social, emotional, or financial) or with mental health issues may find this transition difficult. The fetal and infant brain is highly plastic and needs basic sensory, social, and emotional experiences and protection against toxic stress to develop properly [1, 2]. Infants develop in a responsive environment characterized by nurturing, consistent, and protective interactions with adults [3] and children exposed to neglect or abuse during pregnancy and the first years of life can experience long-term consequences such as poorer health, attachment problems, developmental problems, mental health issues, and poorer educational outcomes than other children [4–8].

The relationship between mother and infant begins to develop during pregnancy. A mother’s ability to bond with the fetus and newborn child may, however, be restricted by the presence of significant social, emotional, or financial concerns or mental health issues such as depression, anxiety, borderline personality disorder, and schizophrenia. Both depression and anxiety are relatively common mental health issues in women of reproductive age in high-income countries with 11.4% of women for example experiencing perinatal depression [9] and 20.7% having at least one anxiety disorder during pregnancy or the postnatal period [10]. Mental health difficulties can cause significant distress and impaired functioning in the mother which is associated with poorer obstetric outcomes such as preterm labor and low birth weight [11–13]. Experiencing childhood trauma such as neglect and abuse can affect child and adult physical and mental health [14]. Women who have experienced childhood neglect also have a higher risk of neglecting their children [15–17].

Intervening early in life through parenting interventions has increased markedly during the last decade. Systematic reviews and meta-analyses find positive results of parenting interventions on child emotional adjustment and behavior, parenting skills, parent mental health, parental sensitivity, and parent-child relationship [18–24]. Further, studies show that early interventions aimed at disadvantaged families are better economical investments than interventions later in life [25, 26]. Parenting interventions, however, are traditionally offered to the parents after the child is born. Most interventions offered during pregnancy are focused on a specific type of intervention such as mindfulness [27] or yoga [28], or one specific health problem such as obesity [29], diabetes [30], breastfeeding [31], or smoking cessation [32, 33]. The complex relationship between pregnancy, delivery, and mental health issues calls for an interdisciplinary, broad, and comprehensive approach to address the multidimensional processes involved [34]. In other words, families with problems within several domains (e.g., mental health, health, parenting, and social issues) require interdisciplinary interventions starting in early pregnancy [35, 36].

One such intervention is Minding the Baby (MTB). MTB is an attachment-based, interdisciplinary home-visiting intervention aimed at improving development, mental, health, and relationship outcomes in vulnerable families having their first child [37]. Two randomized controlled trials (RCT) of MTB in the USA found that MTB families were more likely to be up-to-date with their pediatric immunizations when the child turned 1, less likely to be referred to Child Protective Services, had lower rates of rapid subsequent childbearing, and were more likely to have securely attached infants, improved reflective functioning, less externalizing behavior, and lower levels of obesity [35–41]. An RCT of MTB in the UK found that MTB reduced behavior problems in children and that it might be effective for attachment security, but found no significant differences for any other outcomes [42].

In Denmark, antenatal care, baby health checkups, and social services are free of charge and provided according to the need of the woman. For women with severe mental health issues, severe social issues, or harmful substance abuse (defined as care groups 3 or 4 according to the Danish health authorities’ recommendations for antenatal care), care is expanded to include more check-ups by a specialist team [43] because they have a higher risk of preterm birth and other pregnancy complications [44–46]. A small RCT of an early interdisciplinary and intersectoral perinatal parenting intervention for at-risk pregnant women delivered by midwives and health nurses has been conducted in Denmark, but results are not yet published [47]. It remains unclear if early interdisciplinary intervention is effective when offered to pregnant women with mental health and/or health problems in a European setting with a relatively high level of standard care.

This trial aims to assess the effectiveness of an early and coordinated interdisciplinary intervention (the Family Clinic and Municipality - FACAM intervention) offered to pregnant women in vulnerable positions on the mother-child relationship, maternal social functioning, mental health, reflective functioning, well-being, parental stress, and the development and well-being of the child.

For the purposes of this study, we use the wording “pregnant women in vulnerable positions” to describe pregnant women who are at risk of having complications during the antenatal or perinatal period. This is in accordance with the Danish health authorities’ recommendations for antenatal care for care groups 3 and 4 [48] and the “etic” perspective which relates to an external assessment of risk compared to the normative social expectation according to Spiers [49]. Following the National Institute for Health and Care Excellence (NICE) guideline [50] for antenatal care, we use the terms “woman” and “mother.” These should be taken to include people who do not identify as women but who are pregnant. Similarly, where the term “parents” is used, this should be taken to include anyone who has the main responsibility for caring for a baby.

## Methods and design

**Fig. 1:**
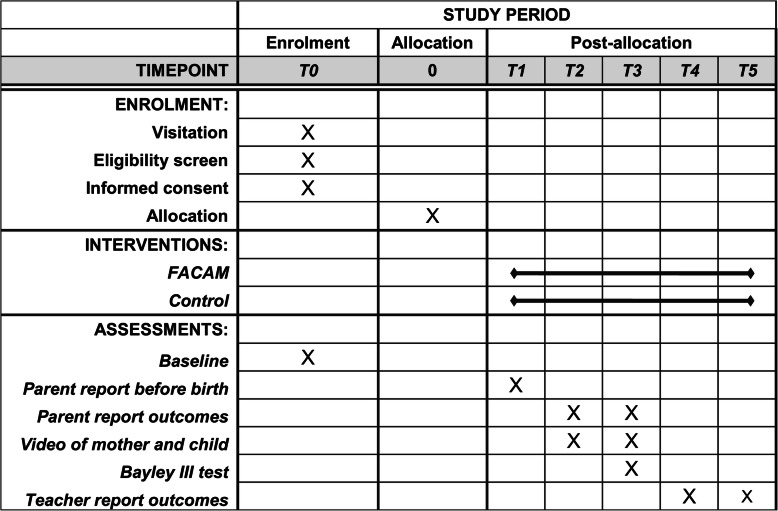
Schedule of enrollment, intervention, and assessments

The study is a prospective, superiority, parallel, 1:1 randomized controlled trial with two study arms: intervention (FACAM) and care as usual (CAU). Pregnant women are randomized to receive either FACAM or CAU. Participants will receive oral and written information on the project and will give written consent to participate in the trial. For participants below 18 consent will also be collected from a parent or guardian. Ethical approval has been obtained from the internal review board at VIVE. The Committee on Health Research Ethics in the Region of Southern Denmark has assessed the protocol and found no need for further approval of the study (Case no. S-20182000-110). The study has obtained approval from the committee of Health Research in the Region of Southern Denmark (journal no. 18/48509). The protocol conforms to the Standard Protocol Items: Recommendations for Interventional Trials (SPIRIT) guidelines (Fig. 1). The final reports of the trial will be written following the Consolidated Standards of Reporting Trials (CONSORT) statement.

### Participants

Participants are pregnant women and their children. To be eligible, women must be included in care group 3 or 4 according to the Danish health authorities’ recommendations for antenatal care [48, 51] and are therefore characterized by having one or more of the following characteristics: (1) severe social problems; (2) unstable family relationships; (3) severe psychological challenges (often caused by childhood trauma, sexual abuse, assault, or neglect); (4) severe previous or present psychiatric disease such as moderate to severe depression, bipolar disease, schizophrenia, or personality disorder; (5) previous or current harmful use of legal or illegal addictive drugs and/or alcohol by the mother and/or father; (6) reduced mental functioning; (7) below age 18; or (8) concern for the mother/child attachment or the parents’ ability to take care of the child.

#### Inclusion and exclusion criteria

Pregnant women at least 15 years old and living in Odense municipality who are characterized as antenatal group 3 or 4 according to the Danish health authorities recommendations, and enrolled to the family clinic at Odense University Hospital are included in the study.

Women are excluded if they fulfill one or more of the following criteria: (1) pregnant with twins, (2) unable to fill out questionnaires in Danish or English, (3) life-threatening illness in parent or child, or (4) previous participation in the FACAM project with an older child. Women are withdrawn from the study if the child is placed in out-of-home care.

### Intervention and comparison

#### Developing the intervention

The FACAM intervention was developed because Odense municipality and the family clinic at Odense University Hospital experienced a need for developing an early, interdisciplinary intervention to support all vulnerable pregnant women and their families. Evaluations of projects carried out in 2012–2017 [52–55] found that a cross-sectional, individual-tailored intervention including practical support (e.g., shopping for the baby, keeping track of appointments, arranging and attending a meeting with the bank) seemed to be acceptable and helpful for the participating women. In 2017, a cross-sectional project group was established including two project leaders, researchers, and professionals from each participating profession/department (the alcohol- and drugs treatment center, health visitors, family therapists, psychologists, social workers, job consultants, and midwives). The group met every month for a year to develop the framework for and content of the intervention. The process also involved leaders from all relevant departments. During the time of development, most of the involved staff interned at the other departments to support a cross-sectional relationship and improve their knowledge about the other departments. Members of the project team also conducted telephone interviews with three pregnant second-time mothers. The interview focused on their experiences of contacts with care professionals during the pregnancy and the first years after birth, and their thoughts about being offered a support person from pregnancy to the start of school. The intervention guidelines were approved by the project steering group.

#### The FACAM intervention

The FACAM intervention consists of assigning a specific support person (FACAM person in the following) to each pregnant woman in the intervention group, a person expected to hold this contact until school-age of the child. The intervention is flexible and tailored to each woman’s individual needs. Theoretically, the intervention builds on theories on how to reduce inequality in health and mentalization theory [56, 57] The idea is that if a pregnant woman or mother has access to practical help and support from a professional she trusts, then she can focus more on parenting the child. There is no strict manual for the intervention but guidelines are specifying different tasks that the FACAM person should address with the participant and how often they should be in contact. The tasks include attending with the participant at healthcare and/or social care visits during pregnancy and after birth or participating in consultations with the midwife, general practitioner, social worker, or job consultant. The tasks also include extra home visits or telephone calls to support the family depending on their needs. Focus for contacts can be practical issues such as reminding the family of inoculations, guiding them on how to register the infant for daycare, but also conversations about the economy, family functioning, health, contraception, childhood upbringing, and mother-child attachment for example. The Danish guidelines are available from the corresponding author. An important task for the FACAM person is to guide and refer the family to appropriate support from the hospital, from child care services, or relevant third sector volunteer organizations. During pregnancy and the first year of the child’s life, the FACAM person can offer up to a total of 47 h of support to the family. Depending on the initial level of concern, all FACAM mothers are also offered either a group-based or an individual-based attachment-based course during pregnancy and the first months of the child’s life. Families with a low level of concern are offered eight 2-h sessions of the Circle of Security Parenting Groups Program (COS-P) from when the child is around 2 months old. Families with a medium or high level of concern are offered up to 50 h of individual sessions with a focus on attachment (including an attachment interview and a focus on mentalization). From when the child is 1 year and until it starts school at age 6, the FACAM person can offer up to 10 h of support to the family each year. It is the intention that the FACAM person is consistent over time. For shorter periods of absence, another FAMKO person will take over the contact with the family if necessary. New FAMKO persons will receive thorough training and support from the other FAMKO persons.

The FACAM person is either a health visitor or a family therapist employed by Odense Municipality. The FACAM persons have participated in a 4-day mentalization-based training and several 1-day courses with a focus on, for example, mental health issues and third sector organizations. They also receive supervision from a clinical psychologist. Furthermore, all health visitors employed at Odense municipality also receive training in the Alarm Distress Baby Scale (ADBB) method. For families where the professionals have a low level of concern, a health visitor is appointed as the FACAM person. They will also act as a regular health visitor for the family. For families with a medium or high level of concern, the FACAM person will be a family therapist. For these families, the regular health visits in the home will be conducted by a health visitor who may not be trained as a FACAM person.

Participants are allowed to receive any other care during the trial. If a participant does not wish to continue with the FACAM intervention or decides to move from Odense municipality, the intervention will be discontinued.

#### Care as usual

Families in the control group receive the usual care that is offered to families with these kinds of challenges. The universal prenatal care offer consists of four to seven midwife consultations, three general practitioner consultations, and two ultrasound scans [58]. Women experiencing at-risk pregnancies receive additional care based on their individual needs including consultations with a social worker, medical doctor, and/or a therapist at the family clinic. The majority of uncomplicated births in Denmark are midwife-assisted hospital births. After hospital discharge, the municipality of residence is informed about the birth, and the family gains access to universal home visiting. Home visits are provided by the municipality within the guidelines issued by the Danish National Board of Health [43]. The standard package offered to all families includes one home visit during pregnancy and 3–5 visits during the first year of life. Municipalities can offer supplementary services such as extra home visits or parenting support interventions to families in need of extra care. All Danish health visitors are registered nurses with 1.5 years of additional specialized training in supporting maternal, child and family health. During the first year of life, all mothers are offered a birth checkup and three well-child checkups with the general practitioner.

The universal care for children 1–6 years old consist of well-child checkups when the child is 2, 3, 4, and 5 years old; a checkup with the dentist when they are 1.5 years old and subsequently every 1–2 years; and a checkup with a health nurse during the first year of school. Families with an increased risk of adversity can be offered home visits by a health visitor at child age 1.5, 2.5, and 3.5.

### Procedures and randomization

**Fig. 2:**
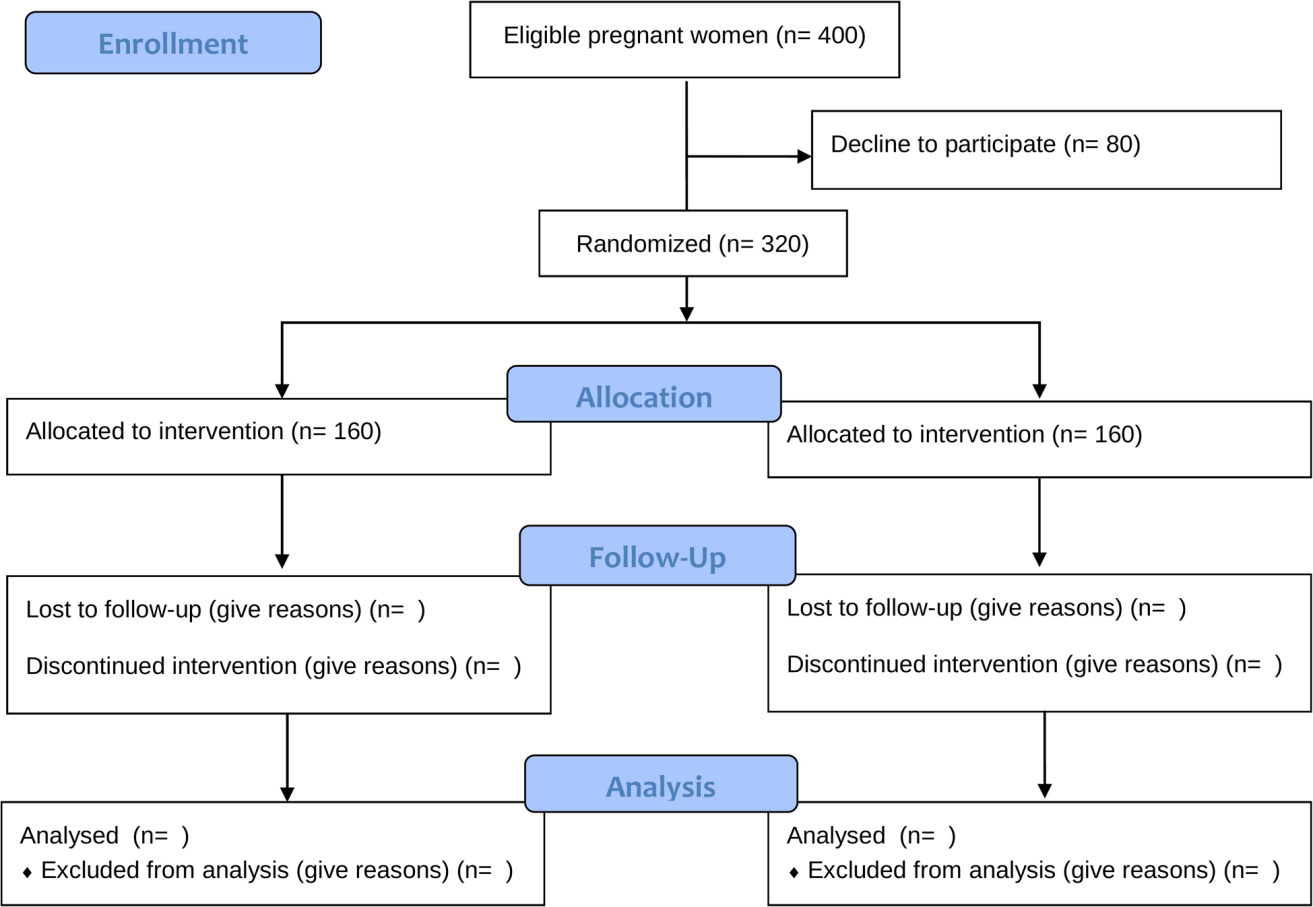
Study flow chart

We will recruit 320 pregnant women—160 to FACAM and 160 to CAU. The flow chart is presented in Fig. 2.

Participants are recruited primarily by the midwives at the family clinic but can also be recruited by research staff. First, the visitation team at the family clinic (comprising a medical doctor, a social worker and/or psychologist/psychotherapist, a midwife, and a secretary) locates eligible pregnant women who meet the criteria of inclusion and adds a study recruitment flyers to their record. At the first consultation at the family clinic, the midwife presents the study, hands out the study folder, and obtains written consent from the participant. If the participant is unsure about participation at the first consultation, she is contacted by the research staff or asked again at the second consultation. At the first consultation, the midwife divides mothers into four categories depending on the level of concern: (1) high level of concern (if there is already a reporting to the child protective services about the family), (2) medium level of concern (if it is likely that there will be a reporting to the child protective services during pregnancy), (3) low level of concern, (3) (if the family is expected to benefit from an attachment-based course but there are otherwise few concerns), or (4) few or no concern about the family. For levels 1 and 2, there is a risk of insufficient parenting competencies and/or child neglect or maltreatment.

When a mother has consented to participate, a research team member registers the participant and sends out the baseline questionnaire. When the participant has filled out the baseline questionnaire, the participant is randomized by a research team member to receive either FACAM or CAU (1:1). The randomization is conducted with the tool “OPEN randomize” in REDCap and the procedure is logged. The randomization sequence was generated by an independent data analyst before recruitment started. Participants are stratified into two groups according to the level of concern assessed by the healthcare professionals which is filled out when participants are invited to participate in the study (levels 1 and 2 are categorized as a high concern, and levels 3 and 4 are categorized as a low concern). When the participant is randomized, the researcher informs the participant and the project coordinator at the municipality about which group the participant is allocated to. The project coordinator at Odense municipality then allocates a FACAM-person to the intervention families prioritizing FAMKO persons with free capacity in the same geographical area. If a group of FAMKO persons with free capacity is under a lot of pressure due to, e.g., sickness, the coordinator will then usually allocate a FAMKO person from another group. After the FAMKO person is allocated, the intervention commences.

The consent form and other related documentation are given to participants are available from the authors on request. The study has no trial steering committee or data monitoring committee as this is not required for behavioral interventions [59, 60].

### Blinding

As the participants are offered extra support in the intervention group, neither participants nor care providers can be blinded. The outcome assessor, coders, and data analysts are blinded to the allocation status. Outcome assessors can only access the ID number and the name and contact information for the participants they are assessing. Coders will only receive the ID number. The intervention and control groups are given two different animal names in the data system to conceal group allocation.

### Data collection

Data are collected through web surveys at four time points: T0, baseline immediately after recruitment; T1, baseline part 2 at gestational week 25; T2, when the infant is 3 months old; and T3, when the infant is 12 months old. The second part of the baseline questionnaire at T1 includes questions about adverse childhood experiences, breastfeeding expectations, and pregnancy reflective functioning which are sent out in a separate questionnaire later in the pregnancy as thoughts about the child is easier to relate to at this time of the pregnancy. At T2, when the child is 3 months old, mothers are also asked to record and upload a 6-min video of mother and child playing together. At T3, when the child is 12 months old, an observational assessment and a 6-min video will be collected at either a home visit or at a municipal location by a research assistant.

Data are collected through a secured online survey database (Research Electronic Data Capture (REDCap)) that is hosted at OPEN Storage, OPEN, Open Patient data Explorative Network, Odense University Hospital, Region of Southern Denmark. REDCap logs data entry and verification. Participants receive an e-mail with a direct link to the questionnaire in e-Boks, a digital mailbox system providing all Danish citizens with a private email account tied to their social security number. Danish public agencies use e-Boks as a secure platform for digital communication with citizens (see www.e-boks.com). Reminders are sent every 3 days by e-mail. If the mothers need help to fill out the questionnaire, they will receive a phone call or help from a member of the research team. Mothers receive a 200 DKK (~ 25 EUR) electronic gift card at each of the three data collections: baseline (T0 and T1), child age 3 months (T2), child age 12 months (T3), child age 24 months (T4), and child age 5 years (T5). The research team will closely monitor the data collection process. Data will be transferred to secure servers hosted by the Agency for Governmental IT Services (Statens IT). The data platform conforms to the international ISO27001 standard on how to manage information security. The trial statistician (MT), the principal investigator (MP), the co-PI (LN), and the senior investigator (EAN) will have access to the full dataset. We do not collect any biological data. Any adverse events will be monitored during the intervention and reported to the PI.

### Measures/outcomes

Socio-demographic measures assessed at T0 - T3 include mother's age, education, occupation, ethnicity, number of children, household status, housing situation, household economy, substance abuse, and breastfeeding expectations. All study measures and outcomes are presented in Table 1.

**Table 1:** The timing of the administration of measures

		T0	T1	T2	T3	T4	T5
Parent measures							
Socio-demographic measures	Age, education, etc.	√		√	√		
Pregnancy reflective functioning	PRFQ-P		√				
Well-being	WEMWBS	**√**		√	√		
Anxiety and depression	HADS	**√**					
Overall health parent and satisfaction	Health and satisfaction	**√**		**√**	√		
Breastfeeding duration				**√**	**√**		
Education and job expectation		√			**√**		
Family budget		**√**		**√**	**√**		
Network	Confidants, support	**√**		**√**	**√**		
Worry		**√**		**√**	**√**		
Use of alcohol, drugs, and medicine use		**√**		**√**	**√**		
Smoking		**√**		**√**	**√**		
Experiences in close relationships	ECR-R	**√**					
PTSD symptoms	PTSD-8		√				
Childhood trauma questionnaire	ACE10		√				
Postnatal depression	EPDS			**√**	**√**		
Being a mother	BaM-13			√			
Parental reflective functioning	PRFQ				**√**		
Parental stress	PSS				**√**		
Anti-conception				**√**	**√**		
Experience with cross-sectional collaboration				**√**	**√**		
Child measures							
Social-emotional development	ASQ-SE2			**√**	**√**		
Child development	ASQ-3			**√**			
Bayley Scales of Infant Development	BSID-III				**√**		
Relationship measures							
Learning activities	Singing, reading				**√**		
Mother and Baby Interaction Scale	MABISC				**√**		
Coding interactive behavior (video)	CIB			**√**	**√**		
Teacher measures							
Social-emotional development	SEAM					**√**	**√**

#### Baseline measures

In addition to the socio-demographic measures, we include the following measures at baseline to assess the initial level and to account for them as possible moderators or confounders in the effect analyses.

Prenatal Parental Reflective Functioning Questionnaire (P-PRFQ) [61] is a 14-item measure of parental reflective functioning of the pregnant woman’s ability to mentalize. The P-PRFQ is an adaption of the PRFQ [62] and consists of three subscales: opacity of mental states (4 items), reflecting on the fetus-baby (3 items), and dynamic of mental states (5 items). Cronbach’s alpha is 0.77 for the total score and 0.69–0.77 for the three subscales. Responses are on a Likert scale ranging from 1 to 7 with three different scalings: (1) high-low where 7 = optimal PRF and 1 = low PRF, (2) low-high where 1 = optimal PRF and 7 = low PRF, and (3) middle where 4 = optimal PRF and 1 and 7 = low PRF. Scores are summed to a total score ranging from 7 to 98.

Hospital Anxiety and Depression Scale (HADS) [63, 64] is a 14 item measure of anxiety and depression. HADS consist of the two subscales “anxiety” and “depression.” The two subscale scores are summed and range from 0 to 21, where low scores indicate less anxiety and depression.

Experiences in the Close Relationship Scale-Short Form (ECR-S) [65] is a 12-item measure of adult attachment consisting of the two subscales “anxiety” (fear of abandonment and a craving for interpersonal closeness) and “avoidance” (fear of intimacy and interpersonal dependency). Each subscale is scored by summing the items and ranges from 1 to 42, where low scores indicate better attachment.

Adverse Childhood Experience (ACE) Questionnaire is a 10-item measure developed for the ACE study to identify childhood experiences of abuse and neglect. Reponses are yes or no. The total score is summed, and the total range is 0–10 where 0 indicates no experience with childhood abuse and neglect.

PTSD-8 [66] is an 8-item measure of posttraumatic stress disorder (PTSD) symptoms, including intrusion, avoidance, and hypervigilance. The total score is the sum of the items and the range is 8–32, where a low score indicates less PTSD.

#### Primary outcome

The primary outcome is maternal sensitivity measured by the Coding Interactive Behavior (CIB) instrument [67] at child age 12 months. The main hypothesis is that mothers in the intervention group will have a higher level of parental sensitivity (a CIB composite) than mothers in the control group. Maternal sensitivity is a subscale of the CIB. The CIB is a global rating system for social interactions that includes 22 parent codes, 16 child codes, and 5 dyadic codes rated on a scale of 1 to 5 which can be aggregated into the following composites: sensitivity, intrusiveness, limit setting, involvement, withdrawal, compliance, dyadic reciprocity, and dyadic negative states. The CIB is coded based on a 6-min mother-infant free play interaction recorded in the home or at another location if preferred by the family. The CIB system has been validated as an assessment measure in multiple studies of mother-child interactions in both normative and high-risk populations and shows stability over time, predictive validity, and adequate psychometric properties [67–71]. Mother-infant interactions are coded by reliable coders blind to treatment allocation. The inter-coder agreement will be calculated on a 10% randomly selected subset of the sample.

#### Secondary outcomes

The parent-child relationship will be measured by the remaining composites of the CIB: intrusiveness, limit setting, involvement, withdrawal, reciprocity, and negative states.

The short version of the Warwick-Edinburgh Mental Well-being Scale (WEMWBS) [72, 73] is a 7-item measure of maternal mental health. A total score is calculated by summing the 7 items and converting the raw score according to a published conversion table. Raw score and converted score range from 7 to 35. A high score indicates better maternal mental health.

Ages and Stages Questionnaire-Social Emotional 2 (ASQ:SE-2) [74] is a measure of child social-emotional development. The ASQ:SE-2 consists of the following seven subscales: self-regulation, compliance, social-communication, adaptive functioning, autonomy, affect, and interaction with people. Items are summed to a total score ranging from 0 to 150 (3 months 15 items) and 0 to 260 (12 months 26 items). A low score indicates better development. Cronbach’s alpha ranges from 0.71 to 0.91. Concurrent validity ranges from 71 to 90%. Sensitivity ranges from 78 to 84%, and specificity ranges from 76 to 98% [75].

Ages and Stages Questionnaire 3 (ASQ:3) [76] is a 30-item measure of child developmental progress. ASQ:3 consists of the following five subscales: communication, gross motor, fine motor, problem-solving, and personal-social. Cronbach’s alpha ranges from 0.67 to 0.85 for the five subscales for the version for children aged 3 months [77]. Items are summed to a total score ranging from 0 to 300 and a low score indicates better development.

Edinburgh Postnatal Depression Scale (EPDS) [78, 79] is a 10-item measure of depression symptoms. Items are summed to a depression total score ranging from 0 to 30. A low score indicates fewer depression symptoms.

Being a Mother (BAM-13) [80] is a 13-item measure of a woman’s satisfaction and experience with being a mother. Items are rated on a 4-point scale (no, hardly ever, no, not very often, yes, some of the time, yes, most of the time). Items are summed to a total score ranging from 0 to 39. A low score indicates higher satisfaction.

Parental Reflective Functioning Questionnaire (PRFQ) [62] is an 18-item measure of parental reflective function or mentalization. The PRFQ consists of three subscales where items are summed and subscale scores range from 6 to 42: (1) Pre-Mentalizing Modes (PRFQ-PM) 6 items (a low score indicates better function), (2) Certainty about Mental States (PRFQ-CMS) 6 items (a high score indicates better function), and (3) Interest and Curiosity in Mental States PRFQ-IC 6 items (a high score indicate better function).

Activities with the child consist of 4 items measuring parent and child interaction through activities such as singing and reading. Items are summed to a total score ranging from 4 to 24. A high score indicates more interaction.

The Parenting Stress Scale (PSS) [81, 82] is an 18-item measure of parenting stress that is rated on a five-point scale (strongly disagree, disagree, undecided, agree, strongly agree). The PSS consists of two subscales: parental stress (items 3, 4, 9, and 10–16) and lack of parental satisfaction (items 1, 2, 5, 6, 7, 8, 17, and 18). When scoring the subscales, LPS items are reversed, and item responses are dichotomized into 0 (strongly disagree and disagree) and 1 (undecided, agree, and strongly agree), and items 2 and 11 are left out. Scores are then summed to subscale scores each ranging from 0 to 9 (PS) and 0–7 (LPS), where a low score indicates less stress and higher satisfaction.

The Mother and Baby Interaction Scale (MABISC) [83] is a 10-item measure of the mother-infant relationship that is rated on a 5-point Likert scale (always, most of the time, occasionally, not often, never). Items are summed to a total score ranging from 0 to 40, where a high score indicates a better relationship.

Bayley Scales of Infant and Toddler Development 3rd Edition - Screening Test (BSID) is a standardized norm-based test widely used to assess child development (Bayley, 2006). The BSID consists of three main subtests; the Cognitive Scale, which includes items such as attention to familiar and unfamiliar objects, looking for a fallen object, and pretend play; the Language Scale, which taps understanding and expression of language, for example, recognition of objects and people, following directions, and naming objects and pictures; and the Motor Scale, which assesses gross and fine motor skills such as grasping, sitting, stacking blocks, and climbing stairs. The cognitive scale assesses memory and problem solving, exploration and manipulation, object relatedness, and sensorimotor development. Raw scores for each subscale are converted into scaled scores (range 1–19, M = 10, SD = 3), and a composite score (M = 100, SD = 15) can be derived from the scaled score for cognitive development, and the sum of the two language-scaled scores. The test will be administered at T3 by trained psychology students supervised by an experienced and trained psychologist.

The mothers will also be asked single items about overall health, life satisfaction, breastfeeding intention, breastfeeding duration, use of birth control, supportive network, loneliness, and how they experience the cross-sectional collaboration between the professionals. If the mother is not employed, she will be asked questions about the indicators for progression toward employment (belief in own skills, mastery of health, and work identity). These items were developed in the employment indicator project [84].

At T4 when the child is 2 years old, the child’s primary teacher in the daycare center will fill out the Social-Emotional Assessment/Evaluation Measure (SEAM) [85]. SEAM is a measure of child social-emotional development consisting of 35 items covering ten benchmarks: empathy, healthy interactions, expression of emotions, regulation of socio-emotional responses, cooperation, sharing and engaging, regulation of attention and activity level, independence, self-image, and adaptive skills. Items are rated on a 4-point scale (very true, somewhat true, rarely true, and never true) plus one column to indicate “concerns” and a column to indicate whether this behavior should be an “intervention goal.” Items are summed to a total score ranging from 0 to 105 (toddler) and a low score indicates better development. In a Danish representative sample of children assessed by the child care teachers Cronbach’s alpha ranges from 0.82 to 0.91 for the toddler version [86].

#### Patient medical records and register data

For both women and children in the intervention and control group, we will ask for consent to retrieve relevant information from social and health registries and medical records from pregnancy until the child is 12 months old. This includes attendance at antenatal care visits, pre-gestational BMI, obstetric complications, mode of delivery, birth weight, birth length, APGAR score, number of reportings to social services, EPDS score at child age 2 months, ADBB score at child age 2 months, and height and weight measurements.

#### Administrative data

When the children are 2 years old, we will retrieve data on all families from Danish administrative data such as the Population Statistics Register, the National Patient Register, the National Prescription Register, the National Health Insurance Service Registry, Oral Health Register, the Education Register and the Danish Rational Economic Agents Model (DREAM). With these data, we can examine outcomes such as vaccinations, child examinations at the general practitioner and dentist, use of social and health care services including emergency visits, use of medication, and information about housing, education, and labor market participation for the parents.

For both intervention and CAU families, we will also collect municipal data on what other interventions the family has been offered (including out-of-home care).

We use participants personal identification numbers to retrieve registered data.

#### Fidelity

During the study period, interdisciplinary meetings with the FACAM persons will be held monthly to support uniformity in the care provided to the families. Furthermore, cross-sectorial meetings with participants from the child-and-youth administration, the alcohol and drug treatment, the family clinic, the FACAM persons, the project leaders, and the researchers are held twice a year to support the cross-sectorial collaboration.

We will collect data on the kind of intervention (e.g., method and provider) and the intensity (number and length of sessions) for both intervention and CAU participants. For the intervention group, the FACAM person will answer a short questionnaire after each visit indicating which kind of support they have offered to the family. Participation in the attachment sessions will also be documented.

### Sample size justification

Power analysis (based on an extension of the dependent *t*-test) was carried out in the design phase to assess the statistical power for testing the primary outcome. The power calculation is based on a meta-analysis of interventions aimed to improve parenting sensitivity [87]. The overall average effect size was 0.44 (standardized mean difference (SMD)), but for randomized trials, the average effect size was 0.33. For normally distributed outcomes and using a two-sided alpha of 0.05, and a power of 80%, we would need 160 participants to detect an effect size of 0.44 and 290 participants to detect an effect size of 0.33. We expect around 15% dropout and therefore aim to recruit 330 participants.

### Data analysis

The primary outcome (CIB parental sensitivity at 12 months) is tested using linear regression with robust standard errors since we are interested in the mean difference between the groups but do not expect a perfectly normal distribution of data. The treatment effect is estimated as a fixed effect using a binary indicator of treatment. A Wilcoxon rank-sum test will be used in the case of severe non-normality. Variables with indications (*p* < 0.1) of the differences between intervention and CAU groups at baseline are used as control variables. Missing data is handled using multiple imputations using all available baseline data. Secondary outcomes are analyzed following the same principles.

Additional exploratory analyses will be carried out in a mixed-effects regression framework. This allows for growth curve estimation. Two-sided tests with 0.05 significance levels are applied throughout.

In addition to the primary analysis, we will perform subgroup analyses to examine the potential differences between subsets of participants. Hence, we analyze the subgroups according to the following characteristics: primiparous or multiparous, education (high school or less versus more than high school), concern about the family (level 1/2 or level 3/4), provider (health visitor or family therapist), adult attachment style (ECR-S), initial trauma level (ACE < 3 or ACE ≥ 3), the initial level of reflective function (lowest 50% versus highest 50%), the initial level of depression or anxiety (clinical or not-clinical level), and attendance (dose).

The primary analysis will be based on the intention-to-treat (ITT) principle, aiming to include all participants in the arm they were originally allocated to irrespective of the amount of treatment received. Missing data is handled using multiple imputations using all available baseline data. Sensitivity analyses will be performed to investigate the potential impact of missing data, in particular, by using a pre-specified conservative multiple imputation strategy and a complete case analysis.

We do not expect that participants will be harmed by the study and therefore have no predefined stopping guidelines and no a priori planned interim analyses.

### Qualitative data

Qualitative data will include focus group interviews and a case study. The focus of the qualitative part is the experiences of the participants and the care providers.

The first aim of the qualitative study is to get insight into how participants and care providers experience the intervention and the interdisciplinary and cross-disciplinary collaboration. We will conduct eight focus groups with care providers and two focus groups with participants. The focus groups will be audio-recorded and transcribed verbatim [88], and data will be analyzed by thematic analysis [89].

The second aim of the qualitative study is to explore how the FACAM intervention is conducted and how participants experience the intervention. Data will be constructed through an ethnographic case study [90] where six participants are followed during as many contacts as possible with their care providers during pregnancy and until the child is 12 months old. Formal as well as informal interviews will be conducted to get an in-depth understanding of the participants’ experiences [91]. The analysis will be based on data from observations, field notes, and audio recordings.

### Protocol amendments

Amendments and changes will be transparently described in the publications following the trial.

### Dissemination of results

Study results will be published in peer-reviewed papers and presented at relevant national and international conferences. Authorship will follow the Vancouver guidelines. To disseminate the results beyond the scientific community, we will publish reports in Danish.

## Discussion

This paper describes in detail the protocol for a randomized controlled trial that aims to investigate the effectiveness of the interdisciplinary FACAM intervention offered to vulnerable pregnant women on the mother-child relationship, maternal social functioning, mental health, reflective functioning, well-being, parental stress, and the development and well-being of the child. The study will provide knowledge about how to best support families with complex problems in early pregnancy and the first years of life.

The participants that we recruit for this study are those that are not traditionally well represented in clinical trials, partly because they have difficulty comprehending uncertainly about allocation and intervention [92]. They have complex problems and challenging life circumstances and are often wary about engaging with social services and may lack the energy to accept extra support. Pregnancy is a window of opportunity for intervening as the pregnant woman usually has a strong motivation to make behavior changes toward greater health for the sake of her baby [93]. Pregnancy may, however, further complicate the relationship with the professionals because the most vulnerable mothers can fear that their child will be removed from their care if they disclose worries or challenges [94]. The process for recruitment and data collection has been tailored to this exact group of pregnant women to increase the chance of recruiting the planned number of participants and retaining them in the study. Examples of tailoring include training of the midwives and incentives for recruiting, making sure the questionnaire is not too long, and offering home visits to collect Bayley III and video data. Some of the women may decline to submit a video recording or participate in the Bayley III assessment as they may be afraid of being judged as a parent. We have developed different possible procedures for video recordings and short informational videos about both the video recording and Bayley III to increase response rates. We will closely monitor both referral, recruitment, and retention rates through the study and accommodate procedures if needed.

When planning the study, we expected that the number of pregnant women classified with a high level of concern (concern groups 1 and 2) and low level of concern (concern groups 3 and 4) by the midwives at the initial visit would be almost equal (160 of each). During recruitment, we have noticed that it is easier to recruit pregnant women with a low level of concern, and consequently, this group is significantly larger than the group of pregnant women with a high level of concern. This is not ideal, and we have tried to accommodate the recruitment process to this. As retention rates are also higher in the group of mothers with a low level of concern, we now expect that this group may end up accounting for 2/3 of the participating mothers. We will monitor data collection on the group of women with a higher level of concern closely and offer them extra support if needed to minimize the risk of attrition within this group.

The trial has been designed in close collaboration with practice and the intervention can be implemented immediately in the usual service if found effective. The intervention is embedded in actual practice and relies on referral to existing services from the FACAM person. The intervention aims to secure that families with a need for extra support get easier access to appropriate interventions when they are supported by a FACAM person that they trust and who knows the system.

To conclude, this is a protocol for a trial aimed at assessing the effectiveness of an early and coordinated interdisciplinary intervention offered to vulnerable pregnant women and their child. We expect that the results will add to the literature on perinatal care and provide useful evidence for future planning and organization of care for vulnerable pregnant women and their families.

## Trial status

This is the second version of the protocol. Recruitment started on October 1, 2018, and is expected to be completed in December 2021.

## Data Availability

The datasets generated and analyzed during the current study are not publicly available to protect participant privacy but are available from the corresponding author on reasonable request.

## Abbreviations

FACAM: Family Clinic And Municipality
CIB: Coding Interactive Behavior
MTB: Minding the Baby
RCT: Randomized controlled trial
CAU: Care as usual
COS-P: Circle of Security parenting groups program
ADBB: Alarm Distress Baby Scale
REDCap: Research Electronic Data Capture
OPEN: Open Patient data Explorative Network
P-PRFQ: Prenatal Parental Reflective Functioning Questionnaire
HADS: Hospital Anxiety and Depression Scale
ECR-S: Experiences in Close Relationship Scale-Short Form
ACE: Adverse childhood experience
PTSD: Posttraumatic stress disorder
WEMWBS: Warwick-Edinburgh Mental Well-being Scale
ASQ:SE-2: Ages and Stages Questionnaire-Social Emotional 2
ASQ:3: Ages and Stages Questionnaire
EPDS: Edinburgh Postnatal Depression Scale
BAM-13: Being a Mother
PRFQ: Parental Reflective Functioning Questionnaire
PSS: Parenting Stress Scale
MABISC: Mother and Baby Interaction Scale
BSID-III: Bayley Scales of Infant and Toddler Development 3rd Edition - Screening Test
SEAM: Social-Emotional Assessment/Evaluation Measure
DREAM: Danish Rational Economic Agents Model
SMD: Standardized mean difference
